# Differential expression of lung-homing chemokine receptors in severe COVID-19: distinct profiles between Alpha and Omicron

**DOI:** 10.1016/j.clinsp.2026.101228

**Published:** 2026-10-30

**Authors:** Fatemeh Sadat Mohammadi, Afsane Fadaee, Alireza Shariati, Saman Soleimanpour, Vahideh Taherian, Ali Mirhosseini, Mehran Mohareri, Narges Valizadeh, Nazila Ariaee, Amir Mohammad Hashem Asnaashari, Seyed Abdolrahim Rezaee

**Affiliations:** a Saveh School of Medical Sciences, Saveh, , Iran; b Immunology Research Center, Inflammation and Inflammatory Diseases Division, Mashhad University of Medical Sciences, Mashhad, Iran; c Department of Internal Medicine, Ghaem Hospital, Faculty of Medicine, Mashhad University of Medical Sciences, Mashhad, Iran; d Department of Microbiology and Virology, School of Medicine, Mashhad University of Medical Sciences, Mashhad, Iran; e Department of Medical Genetics and Molecular Medicine, Faculty of Medicine, Mashhad University of Medical Sciences, Mashhad, Iran

**Keywords:** COVID-19, SARS-CoV-2 variants, ALpha variant, Omicron variant, Chemokine receptors

## Abstract

**Background:**

Severe Coronavirus Disease 2019 (COVID-19), caused by Severe Acute Respiratory Syndrome Coronavirus 2 (SARS-CoV-2), is characterized by excessive pulmonary immune cell recruitment mediated by chemokine – chemokine receptor signaling. Variant-specific differences in lung-homing chemokine receptor expression remain insufficiently defined.

**Methods:**

This retrospective case-control study analyzed Bronchoalveolar Lavage Fluid (BALF) samples from Intensive Care Unit (ICU) admitted patients with severe COVID-19 during the Alpha (hICU-α) and Omicron (hICU-Ο) waves, along with SARS-CoV-2 – negative controls. Messenger RNA expression of CXCR1, CXCR2, CXCR3, CCR2, and CCR6 was quantified by Real-Time quantitative Polymerase Chain Reaction (RT-qPCR). Protein expression of selected receptors was assessed by western blotting in available samples.

**Results:**

CXCR1 expression was significantly increased in hICU-α patients compared with controls (p = 0.02) but not in hICU-Ο patients. CXCR2 expression was elevated in both hICU-α and hICU-Ο groups versus controls (p = 0.008 and p = 0.006, respectively) and was higher in hICU-α than hICU-Ο patients (p = 0.009). CXCR3 expression was increased in both COVID-19 groups compared with controls (p = 0.03 and p = 0.001). CCR6 expression was highest in hICU-Ο patients compared with hICU-α and controls (p = 0.04 and p = 0.001). Increased CXCR2 and CXCR3 protein expression was confirmed in hICU-Ο patients.

**Conclusions:**

Severe COVID-19 is associated with variant-specific alterations in lung chemokine receptor expression, highlighting differential immune trafficking patterns during Alpha and Omicron waves.

## Highlights

• Chemokine receptor expression plays a key role in pulmonary immune cell recruitment in severe COVID-19.
• CXCR1, CXCR2, CXCR3, and CCR6 expression in the lung are associated with disease severity in ICU-admitted COVID-19 patients.
• CXCR2 and CCR6 expression differ between Alpha and Omicron waves, suggesting variant-specific immune responses.
• CCR6 and CXCR2 may help distinguish immunological differences between SARS-CoV-2 variants at the pulmonary level.

## Introduction

Severe Acute Respiratory Syndrome Coronavirus 2 (SARS-CoV-2), first identified in late 2019, rapidly spread worldwide and caused Coronavirus Disease 2019 (COVID-19), a clinical syndrome frequently complicated by Acute respiratory distress syndrome (ARDS).^1^^,^^2^ Since the beginning of the pandemic, successive waves driven by SARS-CoV-2 variants of concern including Alpha (B.1.1.7) and Omicron (B.1.1.529) have posed ongoing challenges to immune protection and clinical management.^3^^,^^4^ Although most infected individuals experience mild disease, a small proportion require hospitalization, and a substantial subset of hospitalized patients progress to critical illness requiring Intensive Care Unit (ICU) admission, often complicated by sepsis and multi-organ dysfunction.^5^, ^6^, ^7^, ^8^ Accumulating evidence indicates that severe COVID-19 is primarily driven by dysregulated host immune responses rather than uncontrolled viral replication alone. Delayed viral clearance and failure to resolve inflammation result in excessive activation and accumulation of immune cells particularly neutrophils, macrophages, and T-lymphocytes within the lungs, leading to immune-mediated tissue damage and organ failure.^9^, ^10^, ^11^

Chemokines and their receptors play a pivotal role in directing leukocyte trafficking and shaping immune responses at sites of infection. Disruption of chemokine–chemokine receptor homeostasis is a hallmark of inflammatory diseases and has been implicated in the pathogenesis of viral respiratory tract infections, including COVID-19.^12^, ^13^, ^14^ Among these pathways, the CXCL8/CXCR1–CXCR2 axis promotes neutrophil recruitment and activation and contributes to Neutrophil Extracellular Trap (NET) formation and inflammatory tissue injury.^15^, ^16^, ^17^ In parallel, the CCL2/CCR2 axis facilitates monocyte and macrophage infiltration into inflamed lung tissue, while the CXCL10/CXCR3 axis mediates the recruitment of Th1, natural killer, and cytotoxic T-cells to sites of inflammation.^16^, ^17^, ^18^

CCR6, predominantly expressed on Th17 cells, regulates their migration toward CCL20-rich inflamed tissues. Th17-derived cytokines particularly interleukin-17 amplify inflammatory cascades by inducing the production of TNF-α, IL-1β, IL-6, and chemokines such as CXCL8 and CXCL10, thereby sustaining immune cell recruitment and systemic inflammation.^19^^,^^20^ Despite the identification of multiple clinical risk factors for severe COVID-19, the immunopathological mechanisms underlying disease severity across different viral variants remain incompletely defined. Given the central involvement of lung-homing neutrophils, monocytes, and CD4⁺ T-helper cell subsets in severe disease, improved characterization of their chemokine-guided trafficking may aid in identifying prognostic biomarkers and therapeutic targets. Therefore, this study evaluated the expression of selected chemokine receptors CXCR1, CXCR2, CCR2, CXCR3, and CCR6 in Bronchoalveolar Lavage Fluid (BALF) from ICU-admitted patients with severe COVID-19 during the Alpha and Omicron waves to assess their association with disease severity.

## Materials and methods

### Participants and study design

All participants were recruited from patients referred to university-affiliated hospitals (Ghaem and Imam Reza Hospitals, Mashhad University of Medical Sciences, Mashhad, Iran). In this retrospective case-control study, we enrolled 24 eligible patients with confirmed COVID-19 who were admitted to the Intensive Care Unit (ICU) between April and May 2021 (Alpha wave in Iran), along with 17 ICU-admitted COVID-19 patients hospitalized between November 2021 and February 2022 (Omicron wave in Iran).^21^ In addition, 40 SARS-CoV-2-negative individuals who underwent bronchoscopy for non-COVID-19 respiratory indications and were asymptomatic at the time of sampling were enrolled as the control group.

The protocol was approved by the Ethics Committee of Mashhad University of Medical Sciences, (IR.REC.MUMS.1401.042). The study was conducted in accordance with the ethical principles of the Declaration of Helsinki and written informed consent was obtained from all participants prior to enrollment. Confidentiality was strictly maintained: all registry and questionnaire data were anonymized prior to analysis, personal identifiers were removed, and access to raw data was restricted to the research team. Strict inclusion criteria were applied to minimize potential confounding factors. For COVID-19 patients, inclusion criteria comprised a positive SARS-CoV-2 RT-PCR test, age between 17- and 75-years, absence of diabetes mellitus, no documented cardiovascular or autoimmune diseases, and no prior vaccination against SARS-CoV-2 at the time of hospital admission. The control group met identical criteria, except for a confirmed negative SARS-CoV-2 PCR result.

### Sample size

This retrospective study included all available ICU-admitted patients with COVID-19 during the Alpha and Omicron waves. A formal sample size estimation was also performed to assess whether the available cohort provided sufficient statistical power. Based on the observed variation in chemokine receptor expression, measured using ΔCt values, and the pooled standard deviations, the effect size (Cohen’s *f*) for a primary outcome such as CCR2 expression was estimated to be 0.47, representing a large effect. For a one-way comparison among three independent groups, with a significance level of α = 0.05 and a desired power of 1−β = 0.80, the required sample size was approximately 12 participants per group.^22^^,^^23^ The actual sample sizes, comprising 24 Alpha-wave ICU patients (hICU-α), 17 Omicron-wave ICU patients (hICU-O), and 40 control subjects, exceeded the calculated minimum required to detect large effect sizes.

### Collection of bronchoalveolar lavage fluid (BALF)

At the discretion of the attending pulmonologist and internist, bronchoscopy with Bronchoalveolar Lavage (BAL) was performed on the first day of ICU admission for COVID-19 patients, as well as in SARS-CoV-2–negative subjects attending respiratory clinics, in accordance with inclusion and exclusion criteria. During bronchoscopy, sterile saline was instilled into a distal bronchus and gently aspirated. Approximately 30 mL of recovered BALF was collected and initially sent for bacteriological and cytological evaluation. Immediately after processing, ∼5 mL of BALF was transferred into sterile Falcon tubes for molecular analyses including quantitative Real-Time PCR (RT-qPCR), and Western blotting and promptly transported to the Inflammation and Inflammatory Diseases Division of the Immunology Research Center, Mashhad University of Medical Sciences.

### RNA extraction, cDNA synthesis, and gene expression measurements

BALF samples were centrifuged at 6000 rpm, and the resulting cell pellets were resuspended in 350 µL Phosphate-Buffered Saline (PBS). Subsequently, 300 µL of the suspension was mixed with 700 µL of TriPure reagent (Roche, Germany) and stored at −70 °C until RNA extraction. Total RNA was extracted using the High Pure RNA Isolation Kit (Roche, Germany) according to the manufacturer’s instructions and reverse-transcribed into complementary DNA (cDNA) using the RevertAid™ First-Strand cDNA Synthesis Kit (Thermo Fisher Scientific, USA).

Primer and probe sequences were designed using Beacon Designer software (PREMIER Biosoft International, CA; version 7) and are presented in Table 1.^24^, ^25^, ^26^ β2-Microglobulin (β2M) was used as the housekeeping gene for normalization of CXCR1, CXCR2, CXCR3, CCR2, and CCR6 expression. RT-qPCR was performed using the TaqMan method on a Rotor-Gene Q6000 real-time PCR system (Qiagen, Hilden, Germany) with Universal TaqMan Master Mix (Takara, Otsu, Japan). Relative gene expression levels were calculated using the 2^⁻ΔΔCT^ method with Rotor-Gene software (Qiagen, Hilden, Germany).

**Table 1:** Oligonucleotide primer sets for RT-qPCR, TaqMan method.

Genes	Accession number	Sequence (5′→3′)
CXCR1	NM_000634.3	**Forward:** 5′- GCTGTTAAGTCACTCTGATCTCTG-3′
		**Reverse:** 5′- GTCCTCTTCAGTTTCAGCAATGG-3′
		**Probe:** 5′- AGCTCCTACTGTTGGACACACCTG GC-3′
CXCR2	NM_001557.4	**Forward:** 5′-TCTTCTGGAGGTGTCCTACAGG-3′
		**Reverse:** 5′ -GAAATCTTCAAAGCTGTCACTCTCC-3′
		**Probe:** 5′-AAAGCCCAGCGACCCAGTCAG GAT-3′
CXCR3	NM_001504.2	**Forward:** 5′-GTGAGTGACCACCAAGTGC-3′
		**Reverse:** 5′-CTCGTTTTCTCCATAGTCATAGG-3′
		**Probe:** 5′- AATGACGCCGAGGTTGCCGCC-3′
CCR2	NM_001123041.3	**Forward:** 5′-CGGCCTGAGTAACTGTGAAAG-3′
		**Reverse:** 5′-CGAAGGCATAGATGATGGGATTG-3′
		**Probe:** 5′-ACTGGACCAAGCCACGCAGGTGACA-3′
CCR6	NM_031409.4	**Forward:** 5′-TGTCACAGAAGTCCTGGCTTTC-3′
		**Reverse:** 5′-TACTTCCTTCTCACACACCACAG-3′
		**Probe:** 5′- ACTGCTGCCTGAACCCTGTGCTCT-3′
β2M	NM_004048.2	**Forward:** 5′-TTGTCTTTCAGCAAGGACTGG-3′
		**Reverse:** 5′-CCACTTAACTATCTTGGGCTGTG-3′
		**Probe:** 5′-TCACATGGTTCACACGGCAGGCAT-3′

CXCR, C-X-C motif Chemokine Receptor; CCR, C—C chemokine Receptor, β2-M, β2-Microglobulin.

### Western blotting analysis

Protein-level validation was performed for selected chemokine receptors based on predefined criteria, including statistically significant differential gene expression across study groups, established relevance to COVID-19-related pulmonary inflammation, and feasibility of protein extraction from available BALF samples. Accordingly, CXCR2 and CXCR3 were selected for western blot analysis. Due to insufficient protein yield from BALF samples collected from hICU-α, protein analyses were restricted to samples from hICU-Ο patients and the control group. Protein expression was analyzed in [n = 3] hICU-Ο patients and [n = 3] SARS-CoV-2–negative control subjects, and densitometric quantification was performed using independent biological replicates from these samples.

Proteins were separated by 12% SDS – Polyacrylamide Gel Electrophoresis (SDS-PAGE) (Bio-Rad, USA) and transferred onto Polyvinylidene Fluoride (PVDF) membranes (Bio-Rad Laboratories, CA, USA). Membranes were blocked with 5% bovine serum albumin (BSA; Sigma-Aldrich, MO, USA) in 0.1% Tween-20 for 1 h at room temperature and incubated with primary antibodies against CXCR2, CXCR3, and β-actin (Abcam, UK). After washing with Tris-Buffered Saline containing 0.1% Tween-20 (TBST), membranes were incubated with HRP-conjugated goat anti-rabbit IgG secondary antibody (Abcam, UK). Protein bands were visualized using Enhanced Chemiluminescence (ECL).

### Statistical analysis

Statistical analyses were performed using SPSS software version 11.5 (SPSS Inc., Chicago, USA) and GraphPad Prism version 7 (GraphPad Software, Inc.). Continuous variables are presented as mean ± Standard Error of the Mean (SEM). Normality of quantitative variables was assessed using the Shapiro-Wilk test. As most variables did not follow a normal distribution, nonparametric statistical tests were applied. For comparisons involving three independent groups (hICU-α, hICU-Ο, and control), the Kruskal-Wallis test was used. When a significant overall difference was detected, post-hoc pairwise comparisons were performed using the Mann-Whitney *U test* with adjustment for multiple comparisons, where appropriate. For comparisons between two independent groups (hICU-Ο vs. control), including western blot densitometry analyses, the Mann-Whitney *U test* was applied. Categorical variables were analyzed using the chi-square test. All statistical tests were two-sided, and a p-value ≤0.05 was considered statistically significant.

## Results

### Subjects

The study population consisted of 24 patients with COVID-19 admitted to the ICU during the Alpha wave, with a mean age of 59.25 ± 3.20 years. 17 ICU-admitted COVID-19 patients during the Omicron wave, with a mean age of 65.11 ± 2.61 years, and 40 SARS-CoV-2–negative individuals undergoing routine bronchoscopy as controls with a mean age of 58.55 ± 1.54 years (Table 2). No statistically significant differences were observed among the three groups with respect to age or sex distribution.

**Table 2:** Demographic characteristics of the study subjects.

Variables		hICU-α	hICU-O	Control	p-value
Age		59.25 ± 3.20	65.11 ± 2.61	58.55 ± 1.54	0.19^a^
Gender	Male	17	9	20	0.16^b^
	Female	7	8	20	

a Age comparison was performed using the Kruskal-Wallis test.

b Gender distribution was compared using the chi-square test. There were no statistically significant differences in age or gender among the study groups; p-value of <0.05, was considered statistically significant.

### The CXCR1 and CXCR2 expression

The mean relative mRNA expression of CXCR1 in hICU-α, hICU-Ο, and control groups was 10.71 ± 3.33, 7.46 ± 3.76, and 2.22 ± 0.44, respectively. CXCR1 expression was significantly higher in hICU-α patients compared with controls (p = 0.02), whereas no significant difference was detected between hICU-Ο patients and the control group (Fig. 1A). The mean relative expression of CXCR2 was 2.82 ± 2.03 in the control group, 21.87 ± 6.97 in hICU-α patients, and 4.18 ± 4.16 in hICU-Ο patients. CXCR2 expression was significantly increased in hICU-α patients compared with both hICU-Ο patients (p = 0.009) and controls (p = 0.008). In addition, CXCR2 expression was significantly higher in hICU-Ο patients than in controls (p = 0.006) (Fig. 1B).

**Fig. 1:**
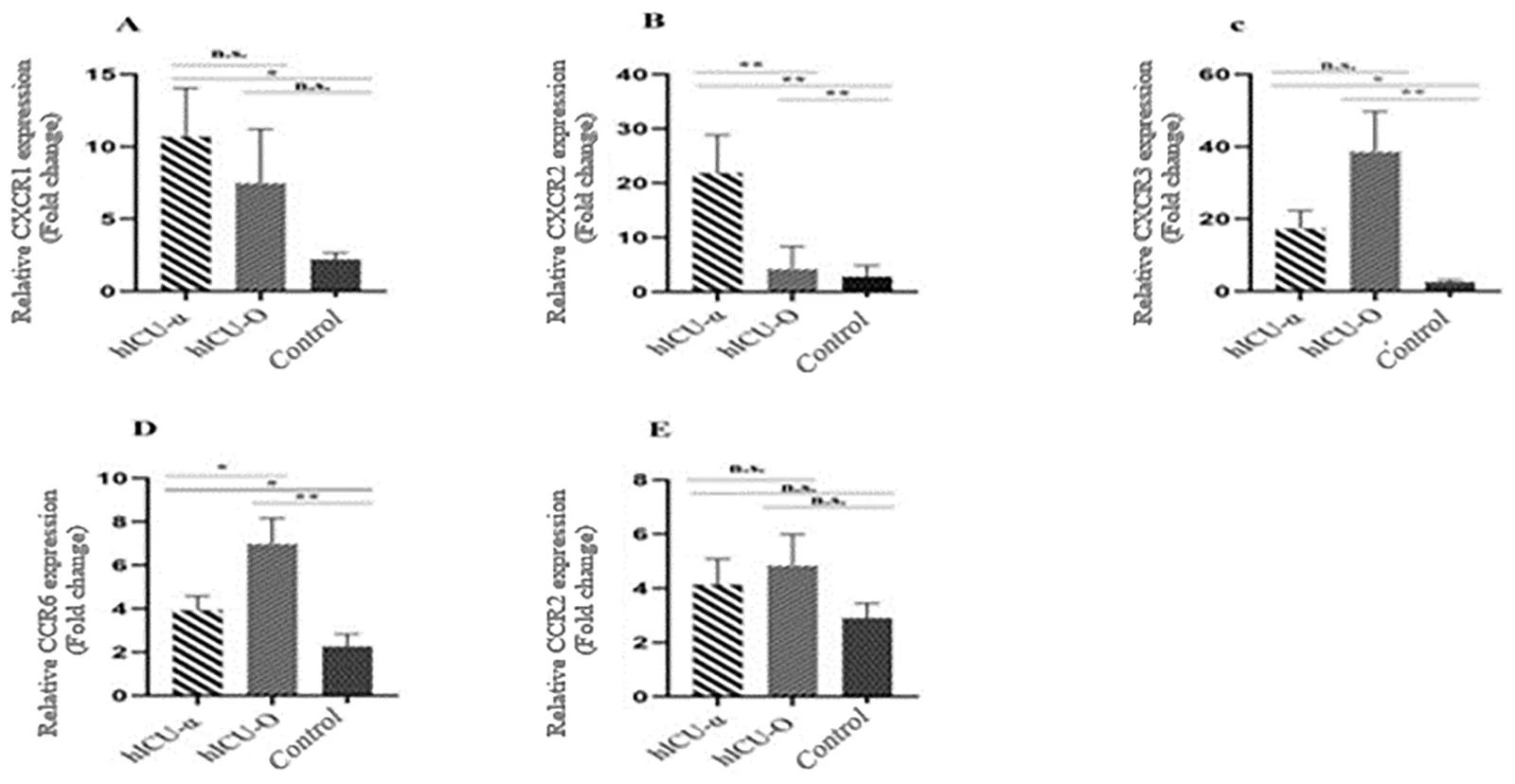
**Relative mRNA expression of CXCR1, CXCR2, CXCR3, CCR6, and CCR2 in Bronchoalveolar Lavage Fluid (BALF) cells.** The relative mRNA expression levels of CXCR1 (A), CXCR2 (B), CXCR3 (C), CCR6 (D), and CCR2 (E) were quantified by RT-qPCR in BALF cells obtained from ICU-admitted COVID-19 patients during the Alpha wave (hICU-α), ICU-admitted COVID-19 patients during the Omicron wave (hICU-Ο), and SARS-CoV-2–negative control subjects (Control group). Gene expression levels were normalized to β2-Microglobulin (β2M), and calculated using the 2^⁻ΔΔCt^ method. Comparisons among the three groups were performed using the Kruskal-Wallis test, followed by Mann-Whitney *U tests* for pairwise comparisons where appropriate. Data are presented as mean ± SEM. NS, non-significant; *p < 0.05; **p < 0.01.

### The CXCR3 expression

The mean relative expression of CXCR3 was 17.39 ± 4.91 in hICU-α patients, 38.55 ± 11.16 in hICU-Ο patients, and 2.61 ± 0.56 in the control group. CXCR3 expression was significantly increased in both hICU-α and hICU-Ο groups compared with controls (p = 0.03 and p = 0.001, respectively) (Fig. 1C). No statistically significant difference in CXCR3 expression was observed between hICU-α and hICU-Ο patients (p > 0.05).

### The CCR6 and CCR2 expression

The mean relative expression of CCR6 was 6.97 ± 1.19 in hICU-Ο patients, 3.94 ± 0.65 in hICU-α patients, and 2.27 ± 0.56 in controls. CCR6 expression was significantly higher in hICU-Ο patients compared with both hICU-α patients (p = 0.04) and controls (p = 0.001). Additionally, CCR6 expression in hICU-α patients was significantly increased compared with controls (p = 0.01) (Fig. 1D). The mean relative expression of CCR2 was 4.14 ± 0.94 in hICU-α patients, 4.83 ± 1.16 in hICU-Ο patients, and 2.91 ± 0.53 in controls. No statistically significant differences in CCR2 expression were observed among the study groups (p > 0.05) (Fig. 1E).

### Protein expression levels

Increased CXCR2 mRNA expression observed in hICU-Ο patients compared with controls was confirmed at the protein level by western blot analysis (Fig. 2A). Similarly, CXCR3 protein expression was significantly higher in hICU-Ο patients than in the control group (Fig. 2B). Protein-level analyses were restricted to hICU-Ο and control samples due to insufficient protein yield from BALF samples collected during the Alpha wave.

**Fig. 2:**
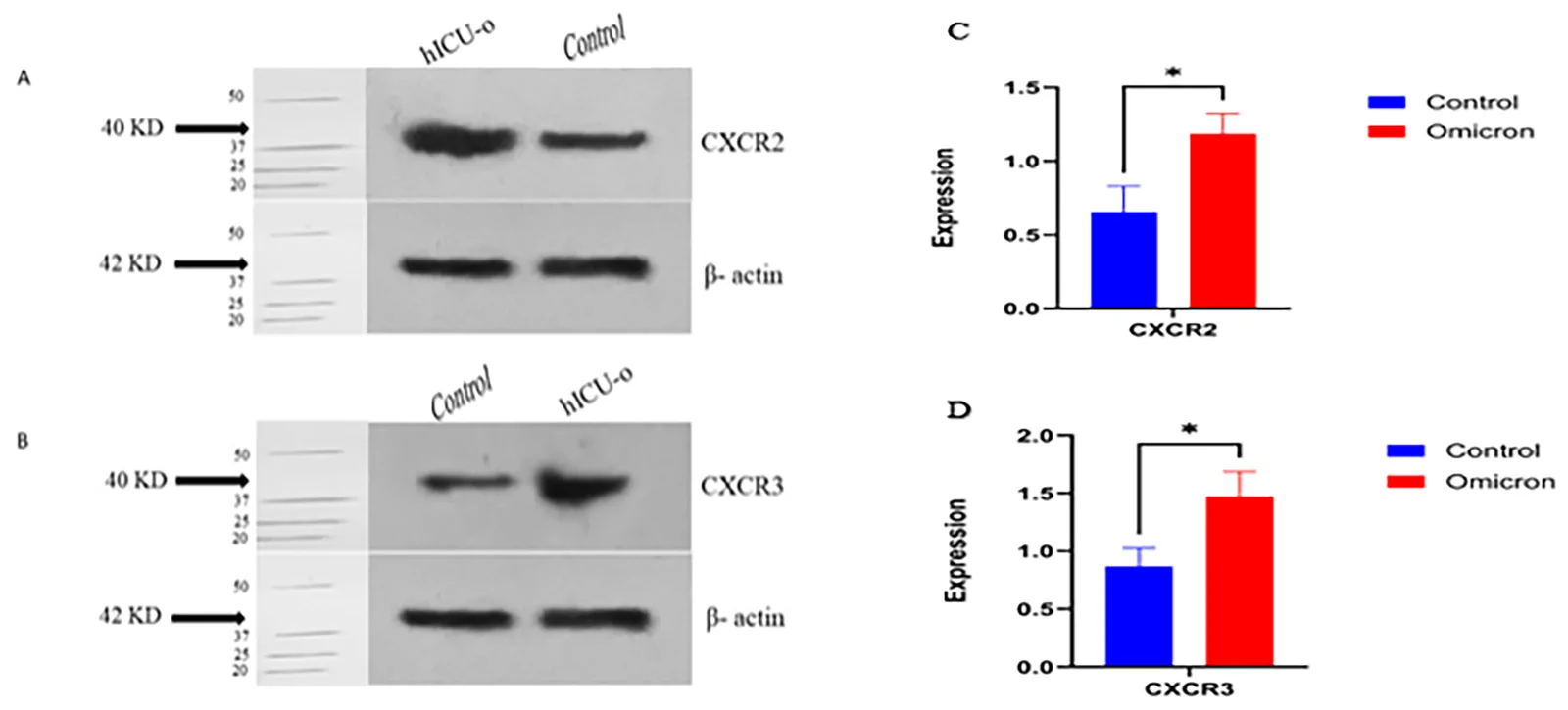
**CXCR2 and CXCR3 protein expression in Bronchoalveolar lavage Fluid (BALF) Cells by western blotting.** Western blot analysis was performed using [n = 3] hICU-Ο patients and [n = 3] SARS-CoV-2–negative controls. (A‒B) Representative western blot images showing CXCR2 and CXCR3 protein expression in hICU-Ο patients and SARS-CoV-2–negative controls, with β-actin used as a loading control. (C‒D) Quantitative densitometric analysis of CXCR2 (C) and CXCR3 (D) protein expression normalized to β-actin. Data are presented as mean ± SEM. *Comparisons between hICU-Ο and control groups were performed using the Mann-Whitney *U test*. p < 0.05 was considered statistically significant. Densitometric analysis of immunoreactive bands revealed significantly higher CXCR2 and CXCR3 protein expression in hICU-Ο patients compared with controls (Mann-Whitney *U test*, p < 0.05).

Quantitative densitometric analysis of western blot bands normalized to β-actin demonstrated a significant increase in CXCR2 protein expression in hICU-Ο patients compared with SARS-CoV-2–negative controls (Mann-Whitney *U test*, p < 0.05; Fig. 2C). Similarly, CXCR3 protein expression was significantly elevated in hICU-Ο patients relative to controls, as confirmed by densitometric analysis (Mann-Whitney *U test*, p < 0.05; Fig. 2D). Protein-level analyses were restricted to hICU-Ο and control samples due to insufficient protein yield from bronchoalveolar lavage fluid collected during the Alpha wave.

## Discussion

In this study, we investigated the pulmonary expression of selected chemokine receptors (CXCR1, CXCR2, CXCR3, CCR6, and CCR2) in BALF cells from ICU-admitted COVID-19 patients during the Alpha and Omicron waves, compared with non–SARS-CoV-2 control subjects. Our findings indicate distinct and variant-dependent alterations in lung-homing chemokine receptor expression, reflecting differences in local immune activation rather than direct evidence of prognosis or therapeutic efficacy. Chemokines and their receptors are central regulators of leukocyte trafficking and activation during viral infections.^12^^,^^27^ In severe COVID-19, dysregulated pulmonary immune responses have been associated with excessive recruitment of neutrophils, monocytes, and activated T-cells, contributing to tissue injury and respiratory failure.^10^^,^^28^ Importantly, emerging SARS-CoV-2 variants differ in pathogenicity and host immune interactions, which may translate into variant-specific pulmonary immune signatures.^27^^,^^29^

In present study, CXCR3 and CCR6 mRNA expression was significantly increased in BALF cells from both hICU-α and hICU-Ο patients compared with controls, suggesting enhanced recruitment of activated T-cell subsets to the lungs in severe disease. Previous studies have shown that CXCR3 is highly expressed on activated CD4⁺ and CD8⁺ T-cells in the BALF of severe COVID-19 patients and is associated with elevated pulmonary CXCL10 levels.^27^^,^^30^ Increased CXCL10 in BALF has been linked to prolonged mechanical ventilation, supporting its role as a marker of severe pulmonary inflammation rather than a validated prognostic factor.^31^

Similarly, CCR6 upregulation, particularly pronounced in hICU-Ο patients, likely reflects increased Th17-associated immune responses. CCR6 is uniformly expressed on Th17 cells, which are implicated in IL-17–driven neutrophilic inflammation in COVID-19.^19^^,^^32^ Elevated CCL20 levels in BALF from mechanically ventilated patients further support enhanced CCR6-mediated cell trafficking to inflamed lung tissue.^33^ However, given the cross-sectional nature of our data, these receptors should be interpreted as correlates of disease severity rather than independent prognostic biomarkers.

No significant differences in CCR2 expression were observed between COVID-19 patients and controls. While CCR2–CCL2 signaling has been implicated in monocyte recruitment and cytokine amplification in severe COVID-19,^16^^,^^34^ this axis is also activated in other pulmonary conditions, including tuberculosis and lung malignancies.^35^^,^^36^ The inclusion of controls undergoing bronchoscopy for non-malignant pulmonary conditions may have attenuated COVID-19 specific differences in CCR2 expression, underscoring a key limitation in interpretability. Thus, CCR2 does not appear to be a robust discriminator of COVID-19–specific lung pathology in this cohort. CXCR1 and CXCR2, key receptors regulating neutrophil recruitment via CXCL8 signaling, demonstrated distinct variant-dependent expression patterns. CXCR1 expression was significantly increased only in hICU-α patients, whereas CXCR2 expression was elevated in both hICU-α and hICU-Ο groups, with higher levels observed during the Alpha wave. These findings align with prior reports linking severe COVID-19 to sustained neutrophil activation, impaired CXCR2 downregulation, and excessive lung infiltration.^37^^,^^38^ Experimental and clinical studies have shown that neutrophil-driven tissue injury, NET formation, and elastase release contribute substantially to severe COVID-19 pathophysiology.^39^, ^40^, ^41^

Our data suggest that CXCR2 and to a lesser extent CXCR1 reflect heightened neutrophil-associated inflammation in severe disease, particularly during infection with earlier, more pathogenic variants. However, these findings should not be interpreted as evidence of therapeutic target validation in the absence of functional or interventional studies. Strengths of this study include direct analysis of pulmonary immune markers in BALF cells and comparison across SARS-CoV-2 variants in ICU-admitted patients. However, several limitations warrant consideration. First, the retrospective cross-sectional design limits causal and prognostic inference. Second, although the sample size was sufficient to detect moderate-to-large effects, smaller differences such as those related to CCR2 may not have been detectable, and these findings should be interpreted cautiously. Although supported by post-hoc power estimation, this study was not based on a pre-specified sample size calculation and therefore may have been underpowered to detect subtle differences between groups. Third, protein-level analyses could not be performed for hICU-α samples due to insufficient BALF protein yield, limiting variant-level protein comparisons. Finally, the control group included individuals with non-COVID-19 respiratory conditions, which may influence pathways such as CCR2 that are shared across inflammatory lung diseases. These conditions may include other underlying inflammatory processes (e.g., bronchitis or pneumonia), and therefore the control group should be considered a “disease control” rather than a healthy population. Future studies should include larger, prospectively recruited cohorts, integrate protein-level and functional assays, and evaluate a broader panel of chemokine receptors and ligands to further clarify variant-specific immune trafficking mechanisms in severe COVID-19.

## Conclusion

In summary, this study identifies distinct patterns of lung chemokine receptor expression in ICU-admitted patients with severe COVID-19. Increased expression of CXCR1, CXCR2, CXCR3, and CCR6 in bronchoalveolar lavage fluid underscores their association with immune cell trafficking and pulmonary inflammation in critical disease. Notably, differential expression of CXCR2 and CCR6 between the Alpha and Omicron waves suggests variant-specific immune responses in the lung. While these findings indicate that selected chemokine receptors correlate with disease severity and variant-related differences, they remain descriptive in nature. Further functional and longitudinal studies are required to determine their mechanistic roles and to assess their potential clinical relevance.

## Abbreviations

COVID-19, Coronavirus Disease 2019; SARS-CoV-2, Severe Acute Respiratory Syndrome Coronavirus 2; CXCR1, C-X-C motif Chemokine Receptor 1; CXCR2, C-X-C motif Chemokine Receptor 2; CXCR3, C-X-C motif Chemokine Receptor 3; CCR2, C—C motif Chemokine Receptor 2; CCR6, C—C motif Chemokine Receptor 6; CXCL1, C-X-C motif Chemokine ligand 1; CXCL2, C-X-C motif chemokine ligand 2; CXCL10, C-X-C motif Chemokine ligand 10; CXCL8, C-X-C motif chemokine ligand 2; CCL2, C—C motif Chemokine ligand 2; CCL20, C—C motif Chemokine ligand 20, RT-qPCR, Real-Time quantitative PCR.

## Author’s contribution

Conceptualization: SA. R; Data curation: FS. M, Formal analysis: FS. M, Funding acquisition: No funding; Investigation: FS.M, S.S, A.M, M.M; Methodology: FS.M, V.T; Project administration: SA.R; Resources: A.SH, AMH.A; Software: A. Fadaee, N. Ariaee; Supervision: SA.R, AMH.A, A.SH; Validation: SA.R; Visualization: N.V, V.T; Writing-original draft: FS.M, A.F, A.SH, S.S, V.T, A.M, M.M, N.V, N.A, AMH.A, SA.R; Writing-review & editing: FS.M, A.F, A.SH, S.S,V.T, A.M, M.M, N.V, N.A, AMH.A, SA.R.

## Consent for publication

Not Applicable.

## Ethics approval and consent to participate

The protocol was approved by the Ethics Committee of Mashhad University of Medical Sciences, (IR.REC.MUMS.1401.042). The study was conducted in accordance with the ethical principles of the Declaration of Helsinki and written informed consent was obtained from all participants prior to enrollment. Confidentiality was strictly maintained: all registry and questionnaire data were anonymized prior to analysis, personal identifiers were removed, and access to raw data was restricted to the research team.

## Funding

This research did not receive any specific grant from funding agencies in the public, commercial, or not-for-profit sectors.

## Data availability

The datasets used and/or analyzed in the current study are available from the corresponding author on reasonable request.

## Conflicts of interest

The authors declare no conflicts of interest.
